# Ischemia during exercise stress testing, an indication of coronary vasomotor dysfunction?

**DOI:** 10.1016/j.ijcha.2024.101580

**Published:** 2024-12-23

**Authors:** Diantha J.M. Schipaanboord, Tijn P.J. Jansen, Luuk Scherpenhuijzen, Caïa Crooijmans, Aukelien C. Dimitriu-Leen, Pim van der Harst, Tim P. van de Hoef, René van Es, Hester M. den Ruijter, Peter Damman, N. Charlotte Onland-Moret, Suzette E. Elias-Smale, on behalf of the IMPRESS consortium

**Affiliations:** a Laboratory of Experimental Cardiology, University Medical Center Utrecht, Utrecht University, Utrecht, the Netherlands; b Department of Cardiology, Radboud University Medical Center, Nijmegen, the Netherlands; c Department of Cardiology, Division Heart and Lungs, University Medical Centre Utrecht, Utrecht University, Utrecht, the Netherlands; d Julius Center for Health Sciences and Primary Care, University Medical Center Utrecht, Utrecht University, Utrecht, the Netherlands

**Keywords:** Exercise stress testing, Coronary function test, Coronary microvascular dysfunction, Coronary artery spasm, ECG

## Abstract

**Background:**

Recently it has been suggested that coronary microvascular dysfunction (CMD) may explain the high false-positive rate of exercise electrocardiographic stress testing (EST). However, patients with angina but non-obstructive coronary artery disease (ANOCA) present with a broader spectrum of coronary vasomotor dysfunction (CVDys), namely coronary artery spasm (CAS), CMD or a combination of both. We aim to investigate the diagnostic value of EST for the entire CVDys spectrum.

**Methods:**

We included patients who underwent coronary function testing (CFT) in the Radboud University Medical Center. For each patient we requested the most recent EST report. ESTs were denoted as positive for ischemia if clinically significant ST-segment depression was detected. We calculated the sensitivity, specificity, positive predictive value (PPV), and negative predictive value (NPV) with 95% confidence intervals for the diagnosis of CVDys and its endotypes.

**Results:**

Of the 105 included patients (87 % women, mean age 57 (±8) years), 22 (21 %) had ischemia during EST. CVDys was diagnosed in 94 patients (90 %), of whom 58 patients had an isolated endotype (CAS: n = 51, CMD: n = 7) and 36 patients had CAS and CMD. Ischemia during EST yielded a high specificity and PPV for CVDys (specificity: 100 % (71.5–100 %), PPV: 100 % (84.6–100 %)), which remained reasonably similar for CAS (specificity: 94.4 % (72.7–99.9 %), PPV: 95.5 % (77.2–99.9 %)), but was lower for CMD (specificity: 85.5 % (74.2–93.1 %), PPV: 59.1 % (36.4–79.3 %)).

**Conclusions:**

Ischemia during EST is highly specific for CVDys in general and can be an indicator for CAS and to a lesser extent for CMD in patients with ANOCA.

## Graphical abstract

Sankey plot of the exercise stress testing and coronary function testing results in 105 patients with angina and non-obstructive coronary artery disease (ANOCA). Created with BioRender.com.

**Figure:**
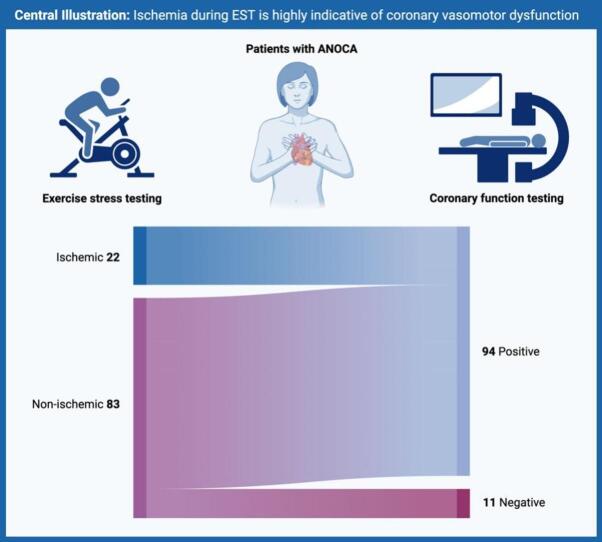

## 1 Introduction

Exercise electrocardiographic stress testing (EST) has long been a first-line screening test for obstructive coronary artery disease (CAD) in patients with angina. [1] The non-invasive and low-cost nature of this test makes it an ideal assessment tool in the early phase of the diagnostic trajectory.[2] However, ESTs exhibit a low sensitivity and a tendency to yield false positive results for obstructive CAD, and with the arrival of new non-invasive stress imaging modalities, ESTs became less important as a screening test for obstructive CAD.[3], [4] This has led to an adjustment of the European Society of Cardiology guideline in which EST was downgraded to a Class 2b recommendation and no longer recommended as the initial test in the diagnosis of stable CAD.[4].

However, obstructive CAD is not the sole cause of symptoms and signs of cardiac ischemia. In 40 to 70 % of the patients with angina undergoing coronary angiography no obstructive CAD is found; the ANOCA patients.[5] With the use of invasive coronary function testing (CFT) it has become clear that a large proportion of these patients have coronary vasomotor dysfunction (CVDys), consisting of coronary artery spasm (CAS) (epicardial or microvascular) and/or coronary microvascular dysfunction (CMD), defined as a reduced coronary flow reserve and/or increased microvascular resistance. We hypothesize that CVDys might be an alternative explanation of a positive EST. A first study evaluating CMD in ANOCA patients showed that the positive predictive value of ischemic ECG changes during EST for CMD was 100 %. However, the negative predictive value was low (33 %).[6] These results suggest that CMD may explain the large false positive rate in EST testing. However, patients with CAS were not included in this study [6], while they represent the largest proportion of CVDys patients (54 % of patients undergoing CFT are diagnosed with isolated CAS, either epicardial and/or microvascular, and 30 % with a combination of CAS and CMD[7]). Therefore, we aim to investigate the diagnostic value of EST for the entire CVDys spectrum, and thereby assess the clinical value of this cheap, non-invasive tool to diagnose CVDys.

## 2 Methods

### 2.1 Study population and data acquisition

We included 244 patients who underwent a CFT in the Radboud University Medical Center, an ANOCA expertise centre, in The Netherlands between February 2019 and November 2020. Patients had persistent angina and no obstructive CAD as underlying cause of their symptoms and were therefore suspected of CVDys and referred for CFT by their treating cardiologist. All patients provided informed consent for the use of their data and for retrieving data retrospectively from previous hospital records. The Medical Ethical Review Committee of the Radboud University Medical Center has approved the research.

Clinical data of these patients, including medical history, cardiovascular risk factors, medication use and CFT result, were collected in a web-based electronic data capture system (Castor EDC, The Netherlands). In this database, data was collected on whether an EST was performed before CFT. We requested the full EST report of the most recent EST per patient by sending a request letter to in total 61 facilities in the Netherlands where the ESTs of the included patients were conducted. All ESTs were carried out according to the ACC/AHA guidelines for exercise testing.[8] The EST reports consisted of the 12-lead ECGs and additional data pertaining to the EST, of which we extracted the heart rates, blood pressures, rate pressure product (RPP) and presence of recognizable symptoms in rest and during exercise.

**Fig. 1:**
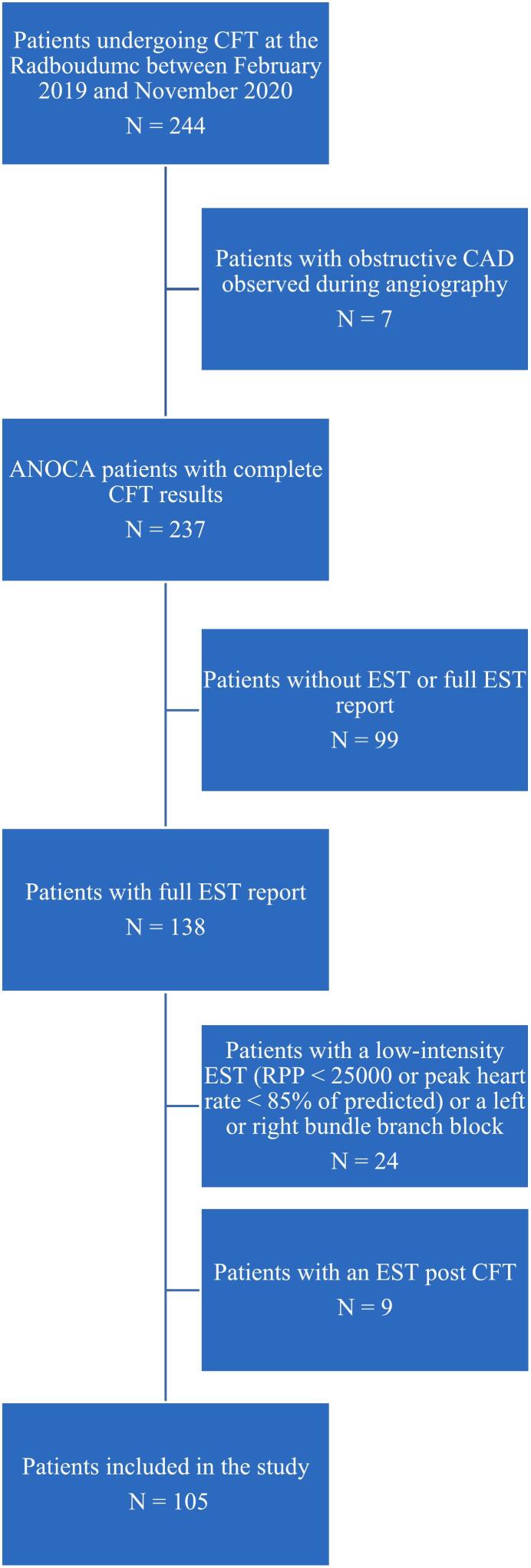
Study flow diagram.

Fig. 1 shows the selection process. We excluded patients 1) in whom obstructive coronary artery disease was observed during angiography (n = 7), 2) who lack a history of EST prior to CFT or in whom an EST report could not be retrieved (n = 99), 3) who had a right or left bundle branch block or a low-intensity EST defined as a RPP below 25,000 in combination with a heart rate at peak exercise less than 85 % of the predicted heart rate (n = 24). After exclusions 105 patients were included in the analysis.

### 2.2 Exercise test analysis

Two experts (TJ and LS) independently assessed the 12-lead ECGs for presence of ischemia using the current criteria for ischemia assessment in EST (ST-segment depression of > 1 mm in men and > 1,5 mm in women in three consecutive heartbeats in two consecutive leads). Both were blinded for CFT outcome. ESTs were denoted as positive if ischemia was detected. All other ESTs were classified as negative.

### 2.3 Outcome measurement

CFT was used as the reference test for the diagnosis of CVDys. CFT was performed according to the previously described protocol.[9] The protocol includes standard diagnostic coronary angiography to rule out obstructive coronary artery disease followed by spasm provocation testing using acetylcholine and assessment of CMD with the bolus thermodilution method using adenosine. Patients undergoing CFT paused the intake of long-acting anti-anginal medication and other vasoactive substances 1–2 days before CFT, to ascertain that vasoreactivity is not influenced by medication when performing spasm provocation testing. Spasm provocation testing was performed by subsequent intracoronary administration of 2, 20, 100 and 200 μg acetylcholine in approximately 1 to 3 min per dose. Each acetylcholine dose was followed by angiography to evaluate the presence of epicardial vasoconstriction. The administration of subsequent doses were omitted in case of >90 % vasoconstriction. CAS was diagnosed according to the Coronary Vasomotion Disorders International Study Group criteria. The presence of anginal symptoms and ischemic ECG changes in combination with > 90 % vasoconstriction on the angiogram following acetylcholine administration yields a diagnosis of epicardial CAS, and symptoms and ECG changes without or with < 90 % vasoconstriction is microvascular CAS. Microvascular function was assessed by two measures; the coronary flow reserve (CFR) and index of microvascular resistance (IMR). These were obtained by positioning of a guidewire with pressure and temperature sensors in the distal part of the left anterior descending artery and repeated intracoronary injection of 3 mL saline boluses in rest and during adenosine induced hyperemia. CMD was diagnosed in case of abnormal CFR (<2.0) and/or abnormal IMR (≥25).

### 2.4 Statistical analysis

Statistical analyses were performed using R (version 4.1.3). We compared baseline characteristics and EST parameters between patients without CVDys, with isolated CAS, with isolated CMD and patients with CAS and CMD. Continuous data are presented as mean with standard deviation or median with interquartile range, where appropriate. We presented categorical data as frequencies and proportions. We calculated the sensitivity, specificity, positive predictive value (PPV), negative predictive value (NPV) with 95 % confidence intervals of EST for CVDys diagnosis in general, CAS diagnosis (both isolated and combined with CMD), CMD diagnosis (both isolated and combined with CAS) and diagnosis of the combined endotype. In a sensitivity analysis, we excluded patients with the combined endotype and calculated the sensitivity, specificity, PPV and NPV with 95 % confidence intervals for CAS diagnosis to investigate if the results are driven by one of the endotypes.

## 3 Results

In total, we analyzed the data of 105 patients with a mean age of 57 (± 8) years of whom 91 (87 %) were women. We included 94 patients with CVDys of whom 36 patients (38.3 %) had the combined endotype (CAS/CMD) while 58 patients had an isolated CVDys endotype (CAS: n = 51 (54.3 %), CMD n = 7 (7.4 %)). CVDys was ruled out in 11 of the included patients.

### 3.1 Baseline characteristics

Table 1 shows the baseline characteristics of the included patients at the time of their CFT, stratified by CFT result. Compared to patients without CVDys, patients with CVDys were slightly older, less often female, less often current or former smokers, had a slightly higher BMI and more often had hypertension. None of the included patients were diagnosed with obstructive CAD between EST and CFT. However, fourteen patients had a history of obstructive CAD at time of EST (CAS only, CMD only and CAS/CMD vs no CVDys: 12 %, 14 % and 19 % vs 0 %, respectively). Baseline characteristics stratified by EST result can be observed in Supplemental Table 1.

**Table 1:** Baseline characteristics of the included patients at the time of their CFT stratified by CFT outcome.

	**No CVDys (n = 11)**	**CVDys (n = 94)**		
		**Isolated CAS (n = 51)**	**Isolated CMD (n = 7)**	**CAS/CMD (n = 36)**
**Age (mean ± SD)**	55,5 ± 7,9	56,8 ± 8,5	60,9 ± 5,0	57,3 ± 8,3
**Females**	10 (91 %)	45 (88 %)	6 (86 %)	30 (83 %)
**BMI (mean ± SD)**	24,8 ± 3,1	27,0 ± 4,2	25,5 ± 3,7	26,8 ± 3,9
**Cardiovascular risk factors**				
Hypertension	1 (9 %)	23 (45 %)	5 (71 %)	21 (58 %)
Hypercholesterolemia	3 (27 %)	17 (33 %)	2 (29 %)	14 (39 %)
Diabetes mellitus	1 (9 %)	6 (12 %)	0 (0 %)	2 (6 %)
Positive family history	4 (36 %)	24 (47 %)	1 (14 %)	16 (44 %)
Smoking	9 (82 %)	22 (43 %)	3 (43 %)	21 (58 %)
**Medication use**				
Beta blockers	2 (18 %)	18 (35 %)	5 (71 %)	9 (25 %)
Calcium channel blockers	7 (64 %)	30 (59 %)	4 (57 %)	27 (75 %)
Long-acting nitrates	1 (9 %)	11 (22 %)	0 (0 %)	9 (25 %)
Nicorandil	0 (0 %)	8 (16 %)	1 (14 %)	9 (25 %)
Anti-platelets	4 (36 %)	23 (45 %)	2 (29 %)	12 (33 %)
Anti-hypertensives	2 (18 %)	23 (45 %)	0 (0 %)	19 (53 %)
Statin	5 (45 %)	28 (55 %)	2 (29 %)	16 (44 %)
**Medical history at time of EST**				
Obstructive CAD	0 (0 %)	6 (12 %)	1 (14 %)	7 (19 %)

BMI = Body mass index; CAD = Coronary artery disease; CAS = Coronary artery spasm; CMD = Coronary microvascular dysfunction; CVDys = Coronary vasomotor dysfunction; CAS/CMD = CAS and CMD combined.

### 3.2 EST results

Table 2 shows the EST parameters of patients with and without CVDys. A positive EST was observed in 22 patients (21 %) and a negative EST in 83 patients (79 %). Interestingly, all patients with a positive EST were diagnosed with CVDys and all patients without CVDys had a negative EST. In total, 23 % of the patients with CVDys had a positive EST. The median time between EST and CFT was 1,8 years (Q1-Q3: 1.0–3.3 years). Patients with CVDys more often had symptoms during EST than patients without CVDys (44 %, 43 % and 56 % vs 27 %, respectively). Furthermore, they had a lower rest heart rate before exercise compared to patients without CVDys (CAS only, CMD only and CAS/CMD vs no CVDys: 77 [67–90] bpm, 80 [75–91] bpm and 79 [73–85] bpm vs 88 [77–95] bpm, respectively), but similar peak heart rate. In addition, the peak diastolic and systolic blood pressure were higher in all CVDys endotypes in comparison to no CVDys (CAS only, CMD only and CAS/CMD vs no CVDys, peak diastolic blood pressure: 82 [74–94] mmHg, 89 [79–96] mmHg and 86 [78–93] mmHg vs 71 [62–83] mmHg, respectively; peak systolic blood pressure: 191 [167–210] mmHg, 201 [180–208] mmHg and 187 [159–207] mmHg vs 170 [148–178] mmHg, respectively).

**Table 2:** Exercise stress test parameters of patients with and without coronary vasomotor dysfunction.

	**No CVDys** **(n = 11)**	**CVDys (n = 94)**		
		**Isolated CAS (n = 51)**	**Isolated CMD (n = 7)**	**CAS/CMD (n = 36)**
**EST-, n (%)**	11 (100 %)	42 (82 %)	6 (86 %)	24 (67 %)
**EST+, n (%)**	0 (0 %)	9 (18 %)	1 (14 %)	12 (33 %)
**Time between EST and CFT, years**	1.5 [1.0–3.1]	2.0 [1.1–4.5]	1.5 [0.9–2.4]	1.8 [0.9–2.8]
**Symptoms during EST, n (%)**	3 (27 %)	22 (44 %)^a^	3 (43 %)	19 (56 %)^b^
**Rest heart rate, bpm**	88 [77–95]	77 [67–90]	80 [75–91]	79 [73–85]
**Peak heart rate, bpm**	155 [147–170]	155 [142–168]	153 [134–157]	148 [139–162]
**Peak diastolic blood pressure, mm Hg**	71 [62–83]	82 [74–94]	89 [79–96]	86 [78–93]
**Peak systolic blood pressure, mm Hg**	170 [148–178]	191 [167–210]	201 [180–208]	187 [159–207]

a Missing value, new total n CAS only = 50. ^b^ Missing values, new total n CAS/CMD = 34. CAS = Coronary artery spasm; CFT = Coronary function test; CVDys = Coronary vasomotor dysfunction; CMD = Coronary microvascular dysfunction; CAS/CMD = CAS and CMD combined; EST = Exercise stress testing; EST+ = Positive EST; EST- = Negative EST.

**Fig. 2:**
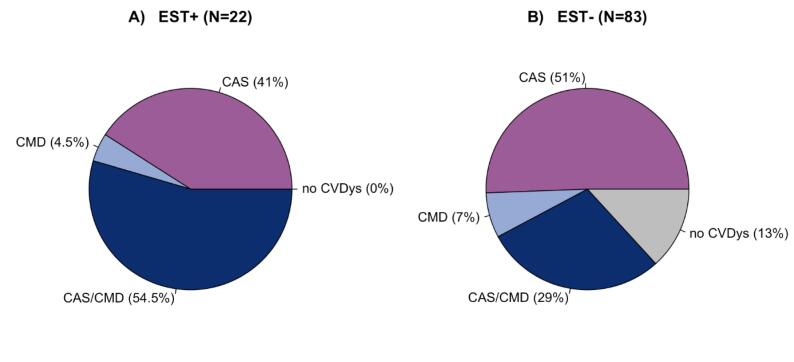
Pie charts of the coronary function test results for patients with A) positive exercise stress testing (EST+), and B) negative exercise stress testing (EST-).

Fig. 2 shows pie charts of the coronary function test results for patients with positive EST (Fig. 2A) and negative EST (Fig. 2B). More than half of all patients with a positive EST (n = 12, 54.5 %) had the combined endotype (Fig. 2A). The majority of the 45.5 % patients with an isolated endotype had CAS (n = 9) and only one patient had CMD. However, also a large portion of patients with CVDys had a negative EST (n = 72, 77 %) (Fig. 2B). Patients with a negative EST were diagnosed with isolated CAS in 51 % (n = 42), in 7 % with isolated CMD (n = 6), in 29 % with a combination of both (n = 24) and 13 % had no CVDys (n = 11). Supplemental Fig. 1 shows the specific underlying endotype for patients with positive or negative exercise stress testing and CAS (epicardial/microvascular spasm) and for patients with CMD (CFR and IMR results).

A positive EST had the highest possible specificity and positive predictive value (PPV), but poor sensitivity and negative predictive value (NPV) to detect CVDys (Sensitivity: 23 %, Specificity: 100 %, PPV: 100 %, NPV: 13 %) (Table 3). This was specifically the case for CAS (isolated or combined with CMD) as underlying CVDys endotype (Sensitivity: 24 %, Specificity: 94 %, PPV: 96 %, NPV: 21 %). For the detection of the combined CVDys endotype, the sensitivity and specificity remained relatively similar to those of CMD (isolated or combined with CAS) (sensitivity: 33 %, specificity: 86 %), with a slightly lower PPV (55 %) and slightly higher NPV (71 %). Sensitivity analysis for the detection of CAS with exclusion of patients with CAS/CMD showed slightly lower sensitivity (18 %) and PPV (90 %), similar specificity (94 %) and slightly higher NPV (29 %) compared to the diagnostic accuracy measures for CAS detection without exclusion of patients with CAS/CMD. The confusion matrices used for calculation of the diagnostic test results in Table 3 are shown in Supplemental Table 2.

**Table 3:** Diagnostic test results of a positive EST for CVDys, CAS, CMD and CAS/CMD diagnosis against no CVDys.

	**Sensitivity, % (95 % CI)**	**Specificity, %** **(95 % CI)**	**PPV, %** **(95 % CI)**	**NPV, %** **(95 % CI)**
**CVDys (n = 105)**	23.4 (15.3–33.3)	100 (71.5–100)	100 (84.6–100)	13.3 (6.8–22.5)
**CAS all (n = 105)**	24.1 (15.6–34.5)	94.4 (72.7–99.9)	95.5 (77.2–99.9)	20.5 (12.4–30.8)
**CMD all (n = 105)**	30.2 (17.2–46.1)	85.5 (74.2–93.1)	59.1 (36.4–79.3)	63.9 (52.6–74.1)
**CAS/CMD (n = 105)**	33.3 (18.6–51.0)	85.5 (75.0–92.8)	54.5 (32.2–75.6)	71.1 (60.1–80.5)
**Sensitivity analysis (exclusion of patients with CAS/CMD)**				
**Isolated CAS (n = 69)**	17.6 (8.4–30.9)	94.4 (72.7–99.9)	90.0 (55.5–99.7)	28.8 (17.8–42.1)

CAS = Coronary artery spasm; CVDys = Coronary vasomotor dysfunction; CMD = Coronary microvascular dysfunction; CAS/CMD = CAS and CMD combined; NPV = Negative predictive value; PPV = Positive predictive value.

## 4 Discussion

We aimed to investigate the diagnostic value of EST for the entire CVDys spectrum in ANOCA patients. The main findings are that 1) all ANOCA patients with a positive EST had CVDys (i.e. excellent PPV), 2) a positive EST seems to be highly indicative of at least CAS as the underlying endotype, and 3) all ANOCA patients without CVDys had a negative EST (i.e. excellent specificity). A positive EST can therefore be very helpful to non-invasively identify patients with a high risk of having CVDys, which is especially valuable in case of CAS as non-invasive options for CAS diagnosis are insufficient. Current non-invasive methods to assess CAS (e.g. the cold-pressor test) have a low sensitivity and are therefore not recommended for diagnosis in patients with suspected CAS.[4].

Performing an EST in patients with angina after ruling out obstructive CAD (i.e. second-line test) can help identify CVDys patients early in the diagnostic process and initiate targeted treatment, potentially averting the need for costly CFT and sparing patients from undergoing invasive testing. Although all patients without CVDys had a negative EST, having a negative EST certainly does not rule out CVDys. Hence, assessment of CVDys via CFT remains a viable consideration in ANOCA patients with negative EST who continue to have symptoms.

This study shows that having CVDys is very likely in patients with a positive EST in whom obstructive CAD is ruled out. This is in line with the previously published study by Sinha et al.[6] in which an PPV of 100 % was observed for CMD diagnosis in ANOCA patients with a positive EST. However, we did not observe a 100 % PPV and specificity for CMD diagnosis in our study. The reason for this can be found in the difference of CMD definition used. We defined CMD as endothelium-independent microvascular dysfunction diagnosed with a CFR < 2.0 and/or IMR ≥ 25, while they defined CMD also as endothelium-dependent microvascular dysfunction (acetylcholine flow reserve ≤ 1.5) in addition to endothelium-independent microvascular dysfunction (CFR < 2.5), which they found to be the strongest predictor of ischemia during EST. Our results for CMD are reasonably comparable with their results for endothelium-independent microvascular dysfunction (sensitivity: 40 %, specificity: 77 %, PPV: 63 %, NPV: 57 %).

Our study demonstrates that EST has a broader clinical utility in the field of ischemic heart disease than obstructive CAD alone. We explored the diagnostic value of EST across the entire CVDys spectrum and our findings underscore the differences in the diagnostic value of EST across distinct CVDys endotypes, with ischemia during EST mainly being an indicator for CAS and to a lesser extent for CMD in our study population. A possible explanation may be that CAS can induce distinct ECG changes detectable during EST, while CMD may exhibit less detectable ECG changes. The exact reason for ischemia during EST in patients with CAS remains unclear. The two mechanisms thought to contribute to CAS are vascular smooth muscle cell hyperreactivity and endothelial dysfunction.[10], [11] In case of vascular smooth muscle cell hyperreactivity and/or endothelial dysfunction, CAS can be provoked during exercise due to the subsequent effects of exercise, such as an increase in circulating vasoactive factors. These temporary spasms of the coronary arteries can lead to supply ischemia. However, in patients with CAS and endothelial dysfunction, the inability of the coronary arteries to adequately dilate can result in a mismatch between the coronary blood flow and increased oxygen demand during exercise and can therefore lead to demand ischemia.[10], [11].

Our study had some limitations. Patients referred for CFT to an ANOCA expertise centre represent a subset of the ANOCA patients, which may explain the high prevalence of CVDys in our study population. Most likely our population consists of patients with more severe complaints, and potentially more severe CVDys. The sensitivity of EST may depend on the severity of CVDys, potentially altering its diagnostic value. Whether EST may also predict the presence of CVDys in an ANOCA population with less severe symptoms needs to be further investigated. Second, the reason to undergo CFT can be based on previous test results, including a previously performed EST. Patients with a positive EST may therefore have had a higher chance of being referred for CFT. In general, the time between EST and CFT may raise the question whether the two tests were done for the same symptoms and whether CVDys was present at time of EST. Given that angina was the indication of all ESTs and none of the patients received a diagnosis for their symptoms (no obstructive CAD diagnosed between EST and CFT), we assumed that the EST and CFT concern the same diagnostic process for the patients in our study. This is not an uncommon assumption, as it has been demonstrated that the healthcare journey of ANOCA patients is generally lengthy.[12] Furthermore, the ESTs were performed with different protocols, which may have led to differences in the ESTs. However, the influence of this on our results will be limited as we excluded the low-intensity ESTs and reassessed the presence of ischemia during EST by two independent experts. Moreover, we were unable to obtain EST reports of 99 patients in total, as these were not performed or no full report was available. This loss was likely random and therefore probably did not introduce bias. At last, we cannot make definitive statements about the diagnostic value of EST for CMD considering the low prevalence of the isolated CMD endotype in our study population. However, as our findings for CMD reasonably align with the endothelium-independent microvascular dysfunction results of a recently published study[6], EST seems to be more indicative of CAS than CMD.

Our results suggest that EST can play a role in the diagnostic pathway of patients with ANOCA. Cross-sectional studies are needed to further identify the diagnostic value of EST for CVDys.

## 5 Conclusions

Ischemia during EST is highly specific for CVDys in our study population and can be an indicator for CAS and to a lesser extent CMD in patients with ANOCA.

## Sources of funding

This project is part of the Dutch Cardiovascular Alliance Consortium IMPRESS (2020B004).

## CRediT authorship contribution statement

**Diantha J.M. Schipaanboord:** Writing – review & editing, Writing – original draft, Visualization, Validation, Software, Project administration, Methodology, Investigation, Formal analysis, Data curation, Conceptualization. **Tijn P.J. Jansen:** Writing – review & editing, Writing – original draft, Visualization, Validation, Resources, Project administration, Methodology, Investigation, Data curation, Conceptualization. **Luuk Scherpenhuijzen:** Writing – review & editing, Validation, Resources, Project administration, Methodology, Investigation, Data curation, Conceptualization. **Caïa Crooijmans:** Writing – review & editing, Conceptualization. **Aukelien C. Dimitriu-Leen:** Writing – review & editing, Resources. **Pim van der Harst:** Writing – review & editing, Supervision. **Tim P. van de Hoef:** Writing – review & editing. **René van Es:** Writing – review & editing, Visualization, Supervision, Methodology, Conceptualization. **Hester M. den Ruijter:** Writing – review & editing, Visualization, Supervision, Methodology, Funding acquisition, Conceptualization. **Peter Damman:** Writing – review & editing, Supervision, Resources, Project administration, Methodology, Conceptualization. **N. Charlotte Onland-Moret:** Writing – review & editing, Visualization, Supervision, Project administration, Methodology, Conceptualization. **Suzette E. Elias-Smale:** Writing – review & editing, Visualization, Supervision, Resources, Project administration, Methodology, Investigation, Conceptualization.

## Declaration of competing interest

The authors declare that they have no known competing financial interests or personal relationships that could have appeared to influence the work reported in this paper.
