# Supplementary material for: Ischemia during exercise stress testing, an indication of coronary vasomotor dysfunction?
Source: Int J Cardiol Heart Vasc. 2024 Dec 23;56:101580. doi: 10.1016/j.ijcha.2024.101580 (PMC11728068; doi:10.1016/j.ijcha.2024.101580)
Supplement: Supplementary Data 1 [file mmc1.docx]

**Supplemental Material**

**Supplemental Table 1**

**Supplemental Figure 1**

**Supplemental Table 2**

**Supplemental Table 1: Baseline characteristics at time of CFT stratified by EST result.**

|  | **EST+ (n=22)** | **EST- (n=83)** |
| --- | --- | --- |
| Age (mean ± SD) | 54,7 ± 7,9 | 57,7 ± 8,2 |
| Females | 19 (86%) | 72 (87%) |
| BMI (mean ± SD) | 26,3 ± 3,7 | 26,6 ± 4,1 |
| **Cardiovascular risk factors** | | |
| Hypertension | 11 (50%) | 39 (47%) |
| Hypercholesterolemia | 7 (32%) | 29 (35%) |
| Diabetes mellitus | 0 (0%) | 9 (11%) |
| Positive family history | 7 (32%) | 38 (46%) |
| Smoking | 11 (50%) | 44 (53%) |
| **Medication use** | | |
| Beta blockers | 7 (32%) | 27 (33%) |
| Calcium channel blockers | 16 (73%) | 52 (63%) |
| Long-acting nitrates | 2 (9%) | 19 (23%) |
| Nicorandil | 4 (18%) | 14 (17%) |
| Anti-platelets | 9 (41%) | 32 (39%) |
| Anti-hypertensives | 13 (59%) | 31 (37%) |
| Statin | 8 (36%) | 43 (52%) |
| **Medical history at time of EST** | | |
| Obstructive CAD | 2 (9%) | 12 (14%) |

BMI = Body mass index; CAD = Coronary artery disease; EST = Exercise stress test; EST+ = Positive EST; EST- = Negative EST. BMI missing: EST+ n=3 (14%), EST- n=4 (5%).

**
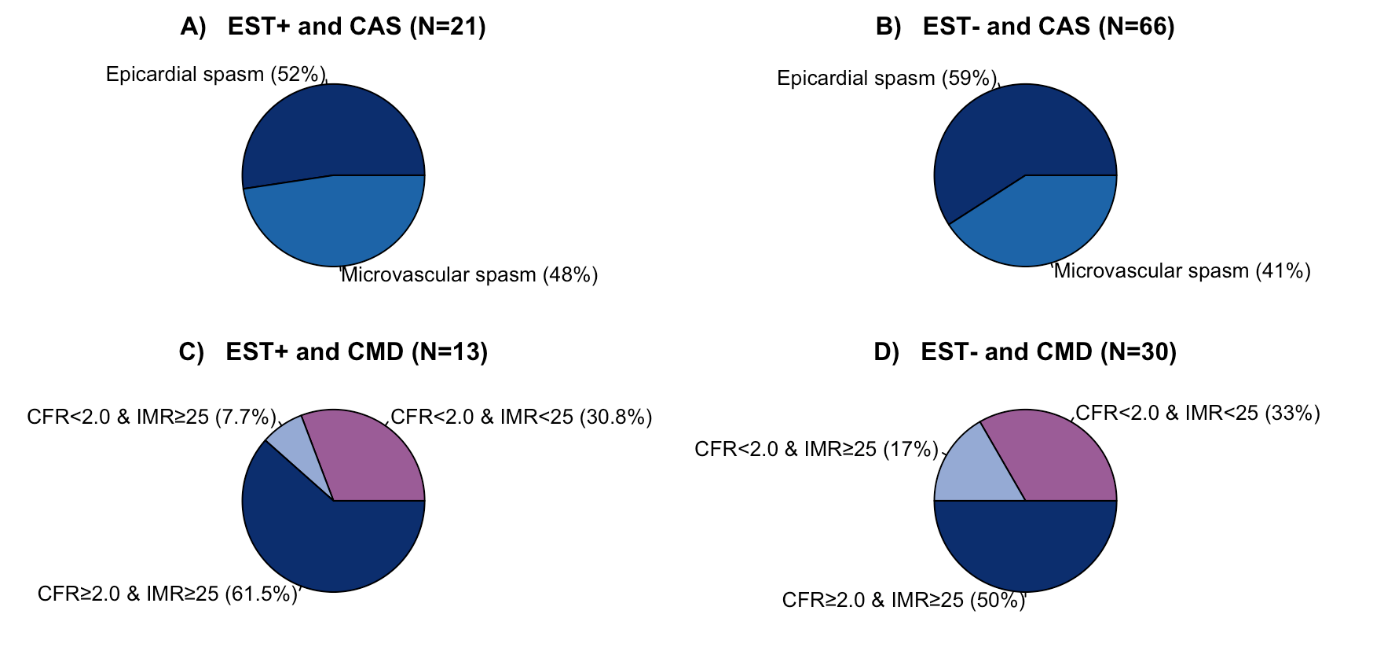
**

**Supplemental Figure 1: Pie charts of the coronary function test results for patients with coronary artery spasm or coronary microvascular dysfunction and positive or negative exercise stress testing.** A) Positive exercise stress testing and coronary artery spasm (EST+ and CAS), B) negative exercise stress testing and coronary artery spasm (EST- and CAS), C) positive exercise stress testing and coronary microvascular dysfunction (EST+ and CMD), D) negative exercise stress testing and coronary microvascular dysfunction (EST- and CMD).

**Supplemental Table 2: Confusion matrices** **used for calculation of the diagnostic test results in Table 3.**

|  | CVDys+ | CVDys- | Total |  |
| --- | --- | --- | --- | --- |
| EST+ | 22 | 0 | 22 | PPV=100% (84,6-100) |
| EST- | 72 | 11 | 83 | NPV=13,3% (6,8-22,5) |
| Total | 94 | 11 | 105 |  |
|  | Se=23,4% (15,3-33,3) | Sp=100% (71,5-100) |  |  |

|  | CAS+ | CAS- | Total |  |
| --- | --- | --- | --- | --- |
| EST+ | 21 | 1 | 22 | PPV=95,5% (77,2-99,9) |
| EST- | 66 | 17 | 83 | NPV=20,5% (12,4-30,8) |
| Total | 87 | 18 | 105 |  |
|  | Se=24,1% (15,6-34,5) | Sp=94,4% (72,7-99,9) |  |  |

|  | CMD+ | CMD- | Total |  |
| --- | --- | --- | --- | --- |
| EST+ | 13 | 9 | 22 | PPV=59,1% (36,4-79,3) |
| EST- | 30 | 53 | 83 | NPV=63,9% (52,6-74,1) |
| Total | 43 | 62 | 105 |  |
|  | Se=30,2% (17,2-46,1) | Sp=85,5% (74,2-93,1) |  |  |

|  | CAS/CMD+ | CAS/CMD- | Total |  |
| --- | --- | --- | --- | --- |
| EST+ | 12 | 10 | 22 | PPV=54,5% (32,2-75,6) |
| EST- | 24 | 59 | 83 | NPV=71,1% (60,1-80,5) |
| Total | 36 | 69 | 105 |  |
|  | Se=33,3% (18,6-51,0) | Sp=85,5% (75,0-92,8) |  |  |

Sensitivity analysis (exclusion of patients with CAS/CMD)

|  | Isolated CAS+ | Isolated CAS- | Total |  |
| --- | --- | --- | --- | --- |
| EST+ | 9 | 1 | 10 | PPV=90% (55,5-99,7) |
| EST- | 42 | 17 | 59 | NPV=28,8% (17,8-42,1) |
| Total | 51 | 18 | 69 |  |
|  | Se=17,6% (8,4-30,9) | Sp=94,4% (72,7-99,9) |  |  |
